# Direct reticular projections of trigeminal sensory fibers immunoreactive to CGRP: potential monosynaptic somatoautonomic projections

**DOI:** 10.3389/fnins.2014.00136

**Published:** 2014-06-05

**Authors:** W. Michael Panneton, Qi Gan

**Affiliations:** Department of Pharmacological and Physiological Science, St. Louis University Medical School, St. Louis, MO, USA

**Keywords:** diving response, autonomic reflex, rostral ventrolateral medulla, caudal ventrolateral medulla, lateral reticular formation, nasal mucosa, anterior ethmoidal nerve

## Abstract

Few trigeminal sensory fibers project centrally beyond the trigeminal sensory complex, with only projections of fibers carried in its sensory anterior ethmoidal (AEN) and intraoral nerves described. Fibers of the AEN project into the brainstem reticular formation where immunoreactivity against substance P and CGRP are found. We investigated whether the source of these peptides could be from trigeminal ganglion neurons by performing unilateral rhizotomies of the trigeminal root and looking for absence of label. After an 8–14 days survival, substance P immunoreactivity in the trigeminal sensory complex was diminished, but we could not conclude that the sole source of this peptide in the lateral parabrachial area and lateral reticular formation arises from primary afferent fibers. Immunoreactivity to CGRP after rhizotomy however was greatly diminished in the trigeminal sensory complex, confirming the observations of others. Moreover, CGRP immunoreactivity was nearly eliminated in fibers in the lateral parabrachial area, the caudal ventrolateral medulla, both the peri-ambiguus and ventral parts of the rostral ventrolateral medulla, in the external formation of the nucleus ambiguus, and diminished in the caudal pressor area. The nearly complete elimination of CGRP in the lateral reticular formation after rhizotomy suggests this peptide is carried in primary afferent fibers. Moreover, the arborization of CGRP immunoreactive fibers in these areas mimics that of direct projections from the AEN. Since electrical stimulation of the AEN induces cardiorespiratory adjustments including an apnea, peripheral vasoconstriction, and bradycardia similar to those seen in the mammalian diving response, we suggest these perturbations of autonomic behavior are enhanced by direct somatic primary afferent projections to these reticular neurons. We believe this to be first description of potential direct somatoautonomic projections to brainstem neurons regulating autonomic activity.

## Introduction

It is well known the trigeminal nerve innervates both cutaneous and mucosal epithelia in the head, as well as the muscles of mastication. Most central projections of the three divisions of the trigeminal nerve are confined to the trigeminal sensory complex (Marfurt, 1981; Marfurt and Rajchert, 1991), but a few primary afferent fibers project beyond this complex. For example, the anterior ethmoidal nerve (AEN), a small nerve of the trigeminal ophthalmic division innervating the anterior mucosa and vestibule of the nose, has numerous extratrigeminal projections. While we have described the central projections of primary afferent fibers in the AEN of rodents to all parts of the spinal trigeminal nucleus after intrathecal injections of an HRP cocktail (Panneton, 1991; Panneton et al., 2006), we also detailed projections into parts of the reticular formation and parabrachial complex which were confirmed recently by others (Hollandsworth et al., 2009; Cavanaugh et al., 2011).

Innervation of the nasal mucosa is via free nerve endings from small diameter fibers (Cauna et al., 1969), many of which contain peptides, notably calcitonin gene-related peptide (CGRP) and substance P (SubP) (Petersson et al., 1989; Silverman and Kruger, 1989; Stjärne et al., 1989; Finger et al., 1990; Silver et al., 1991; Spit et al., 1993; Matsuda et al., 1994, 1998) from trigeminal ganglion neurons (Silverman and Kruger, 1989; Ichikawa et al., 1993; Matsuda et al., 1994; Schaefer et al., 2002). Most of these fibers are sensory in function (Lucier and Egizii, 1989; Wallois et al., 1991, 1992; Sekizawa and Tsubone, 1994, 1996), and many respond as chemoreceptors (Lucier and Egizii, 1989), creating the “common chemical sense” or chemethesis (Cain and Murphy, 1980; Green and Lawless, 1991; Viana, 2011). Moreover, such sensations, including pain, can be elicited from stimulating the human nasal mucosa (Handwerker and Kobal, 1993; Thürauf et al., Cometto-Muñiz and Cain, 1997; 1993; Cometto-Muniz et al., 1998, 2001; Hummel et al., 2003). In addition, it long has been demonstrated that stimulating paranasal areas induces autonomic adjustments (Angell James and de Burgh Daly, 1972; McRitchie and White, 1974; Drummond and Jones, 1979; Panneton, 1990; Gieroba et al., 1994; Kratschmer, 2001) similar to those of the mammalian diving response.

Indeed, stimulating the AEN electrically elicits profound alterations in cardiorespiratory behavior, including an apnea, a dramatic bradycardia, and an increase in arterial blood pressure (Dutschmann and Herbert, 1996, 1997, 1998a; McCulloch et al., 1999a; Rozloznik et al., 2009). Nevertheless, the extratrigeminal pattern of labeling we saw after transganglionic transport of horseradish peroxidase (HRP) molecules in the AEN (Panneton, 1991; Panneton et al., 2006) is remarkably similar to fibers immunoreactive to CGRP found in similar areas of normal rats. We thus wished to determine if the CGRP and SubP found in certain areas of the reticular formation originates in primary afferent fibers of the trigeminal nerve. The loss of fibers immunostained for CGRP in such non-trigeminal reticular targets after unilateral trigeminal rhizotomy suggest that direct reticular projections of trigeminal primary afferent fibers, particularly those from the AEN, are potential monosynaptic projections to reticular neurons regulating heart rate, arterial blood pressure, and perhaps respiration. All of these automatic functions are modulated during the mammalian diving response. We suggest further that signals transmitted through these primary afferent projections coordinate with those relayed from the medullary dorsal horn (MDH) (Panneton et al., 2006) to induce the mammalian diving response. Parts of this data has been published in abstract form previously (Panneton et al., 2005b).

## Materials and methods

Thirteen adult (~275–401 g) Sprague-Dawley male rats were obtained commercially (Harlan, Indianapolis, IN) and used in this study. All protocols were approved by the Animal Care Committee of Saint Louis University and followed the guidelines of the National Institutes of Health Guide for Care and Handling of Laboratory Animals.

Rats were anesthetized with an intraperitoneal injection of a mixture of ketamine/xylazine (60/40 mg/ml) and placed in a stereotaxic unit. The rat's dorsal skin was incised on the cranium, its temporalis muscle retracted laterally, and a 4 × 4 mm window drilled into its left posterior parietal bone. After removing the posterior cerebral cortex on one side by suction, the trigeminal root was visualized posterior to the trigeminal ganglion. A hooked instrument was placed around the root, and the root avulsed with a quick pull on the instrument. The wounds were closed and the rats injected subcutaneously with buprenorphine (0.1 mg/100 gm). The rats were fed soft food for their 8–14 day survival. Their blink reflex was tested postoperatively to insure a complete transection was performed. Seven rats retained reflex activity and are not included in data analysis while those without a reflex were considered further. These rats were perfused through the heart with a solution of 4% paraformaldehyde in phosphate buffer (pH 7.3), their brains and trigeminal ganglia extirpated, and stored in the refrigerator in the fixative with 20% sucrose. After at least 48 h, the brainstems and some ganglia were cut on a freezing microtome at 40 μm and processed for immunohistochemistry with antibodies against calcitonin gene-receptor protein and SubP.

Every third section was washed three times with 0.1 M PB for 10 min, and then in 0.1 M PB with 0.3% triton for at least 5 min. A series of sections were then processed immunohistochemically overnight with antibodies against either CGRP(rabbit anti-CGRP,1:20,000; ImmunoStar Inc., Hudson, WI, USA) or SubP (rabbit anti-SubP, 1:20,000; ImmunoStar, Inc.) in buffer with 0.3% triton on a shaker at room temperature. The following morning, the sections were washed in PB with 0.3% triton and incubated for 1 h in a solution containing goat anti-rabbit immunoglobulin (Sigma-Aldrich Corp., St. Louis, MO, USA) at a dilution of 1:400. The sections then were incubated in Vectastain ABC Elite solution (1:200; Vector Laboratories, Burlingame, CA, USA) for 1 h, washed in three rinses of PB, and reacted with diaminobenzidine dihydrochloride (DAB) intensified with nickel ammonium sulfate for 4–10 min. Hydrogen peroxide (0.06%) catalyzed the reaction. The sections were then rinsed, mounted serially on gelatinized slides and air-dried. They then were counterstained with Neutral Red, dehydrated in alcohols, defatted in xylenes, and coverslipped with Permount.

**Figure 1:**
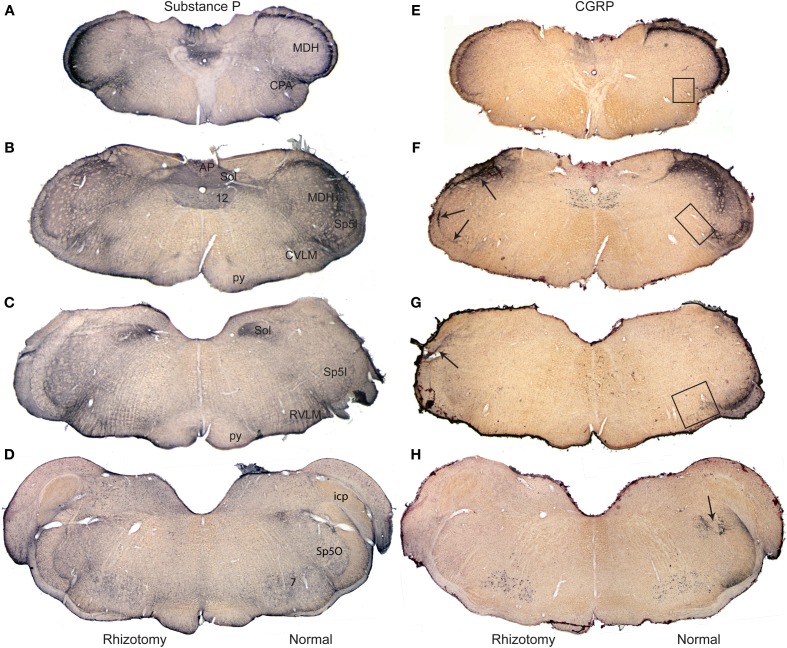
**Composite photomicrographs of brainstem sections cut through caudal (A,E), intermediate (B,F,C,G) and rostral (D,H) parts of the medulla comparing the distribution of immunolabel of substance P (A–D) and CGRP (E–H) after a unilateral trigeminal rhizotomy**. Immunostaining of Substance P, with numerous central and peripheral sources, was little diminished qualitatively after rhizotomy, but that of CGRP, with fewer central neurons, showed up to a 93% reduction in stained fibers after rhizotomy. Note the band of immunolabel seen in the misplaced substantia gelatinosa between the MDH and subnucleus interpolaris **(F)** is nearly eliminated after rhizotomy, while that in the CVLM **(F)** and RVLM **(G)** is greatly diminished. We have shown previously (Panneton et al., 2006) that primary afferent fibers contained within the anterior ethmoidal nerve projects directly to all three areas. Arrows in **(F,G)** point to immunolabel retained after trigeminal rhizotomy; such label mimics the distribution of primary afferent fibers of the glossopharyngeal and vagus nerves. Boxes in **(E–G)** mark areas from which the length of immunolabeled fibers were quantified (Figure 4). Abbreviations: AP, area postrema; CPA, caudal pressor area; CVLM, caudal ventrolateral medulla; MDH, medullary dorsal horn; RVLM, rostral ventrolateral medulla; Sol, nucleus tractus solitarii; Sp5I, subnucleus interpolaris of trigeminal sensory complex; Sp5O, subnucleus oralis of trigeminal sensory complex; py, pyramidal tract; 12, hypoglossal nucleus.

Neurons and fibers immunoreactive to CGRP were visualized with brightfield optics (Nikon E800) and photographed with a digital camera (MicroImager II) and Northern Eclipse Software (Empix, Inc.). Sections of CGRP staining both of whole sections and individual fibers were drawn with a Nikon E600 microscope and Neurolucida software (MicroBrightField, Inc.). Fiber length of CGRP in the caudal pressor area (CPA) (Sun and Panneton, 2002), caudal ventrolateral medulla (CVLM), and rostroventrolateral medulla (RVLM) (Panneton et al., 2006) were drawn and calculated from two sections/case (*n* = 5 cases) from both the normal and rhizotomized sides. Fiber lengths were summed/area and then averaged for both control and experimental sides; combined length from the five cases yielded relative total length. Photographs of older data showing the transneuronal transport of an HRP cocktail applied to the AEN (Panneton et al., 2006) are used to certify the similarity of these projections to those fibers labeled with CGRP. The photomicrographs were standardized using levels, brightness and contrast in Adobe Photoshop CS2 software (v.9) and aligned in Adobe Illustrator software (v.11) for figures. Composite pictures (Figure 1) of whole sections were stitched using functions in Microsoft ICE (Microsoft Image Composite Editor; open source/free—http://research.microsoft.com/en-us/um/redmond/groups/ivm/ice/. All nomenclature and abbreviations are from a stereotaxic rat atlas (Paxinos and Watson, 1998).

## Results

Relatively subtle differences after rhizotomy were noted after immunostaining for SubP when normal and experimental sides were compared (Figures 1A–D). In the trigeminal sensory complex, the immunoreactive fibers in the trigeminal tract were gone on the side with rhizotomy, suggesting effective avulsion of the trigeminal root. The principle trigeminal nucleus lost nearly all of its few SubP reactive fibers in its dorsal half after rhizotomy. Pars oralis of the spinal trigeminal nucleus, which had a dense aggregation of label laterally near the tract and fibers with swellings in its dorsomedial subdivision on the normal side, also lost nearly all label after rhizotomy (Figure 1D). Pars interpolaris of the spinal trigeminal nucleus contained little label even on the normal side (Figure 1C). The paratrigeminal nucleus near the obex lost some intensity of the SubP immunostaining after rhizotomy, but still appeared well-labeled. Similarly much SubP immunoreactivity in laminae I, II, and V of the MDH was lost after root lesions (Figures 1A,B), but some label always persisted. The immunoreactivity in the nucleus tractus solitarii was dense throughout the length of this nucleus, but was unchanged after rhizotomy (Figures 1A–C). It was difficult to detect consistent qualitative changes in reticular targets, including the parabrachial nucleus, RVLM, CVLM, and CPA, since these areas apparently receive input from central as well as peripheral SubP neurons. However, we noted that large SubP fibers leaving the ventral part of trigeminal tract (similar to Figure 3H for CGRP) and entering the RVLM were lost on the rhizotomized side, as well as a subtle decrease of immunostaining surrounding the ambiguus complex rostrally.

**Figure 2:**
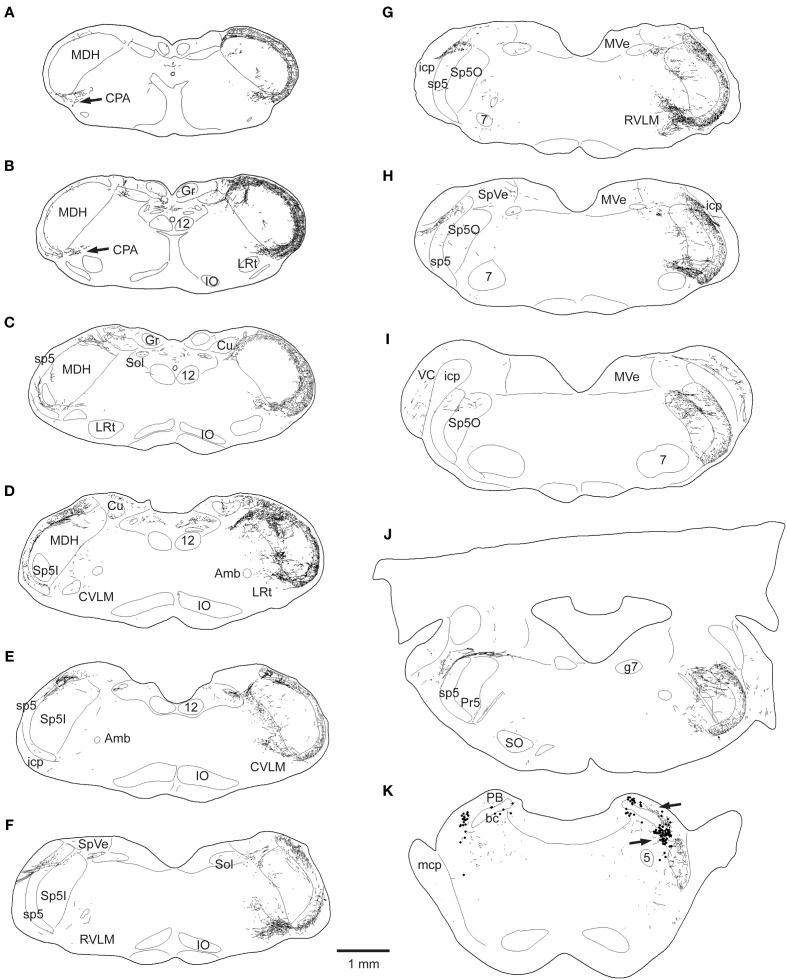
**Line drawings showing the loss of CGRP fibers in the brainstem after unilateral trigeminal rhizotomy**. The normal distribution of CGRP is seen on the right side of these brainstem sections; note its dense distribution in the spinal trigeminal complex, especially in the medullary dorsal horn (MDH) and subnucleus oralis, as well as the CPA **(A,B)**, the CVLM **(D,E)**, peri-ambiguus areas **(D–F)**, the RVLM **(F,G)**, and the parabrachial complex **(K)**. Trigeminal rhizotomy (left side of sections) eliminated almost all fibers in the spinal trigeminal tract and most of the label in the spinal trigeminal nucleus (see text), suggesting its origin in primary afferent fibers with cell bodies in the trigeminal ganglion. Importantly however, CGRP label was particularly diminished in fibers in the lateral reticular formation **(C–J)** areas, also implicating its source in the trigeminal ganglion. All of these reticular areas have been implicated in autonomic function, especially in regard to cardiovascular control. Abbreviations: Amb, nucleus ambiguus; Cu, cuneate nucleus; Gr, gracile nucleus; IO, inferior olivary nucleus; LRt, lateral reticular nucleus; MVe, medial vestibular nucleus; PB, parabrachial complex; PV, principle trigeminal nucleus; SO, superior olivary complex; SpVe, spinal vestibular nucleus; VC, ventral cochlear nucleus; bc, brachium conjunctivum; g7, genu of the facial nerve; icp, inferior cerebellar peduncle; mcp, middle cerebellar peduncle; 5, trigeminal motor nucleus; 7, facial motor nucleus; see Figure 1 for other abbreviations.

Immunostaining for CGRP on the normal side contrasted that for the rhizotomized, experimental side (Figures 1E–H), both in the trigeminal sensory nucleus as well as the reticular formation. Immunoreactivity in the spinal trigeminal tract was lost on the experimental side after rhizotomy (Figure 2). CGRP immunostaining in the principle trigeminal nucleus on the normal side was confined to bursts of meandering fibers in the dorsal half of the nucleus as well as aggregations in its ventromedial tip and along its medial border; both were completely lost after rhizotomy (Figures 2J, 5G,H). Subnucleus oralis showed dense CGRP immunoreactivity along its lateral border with the trigeminal tract as well as more dispersed label in its dorsomedial subdivision on the normal side (Figure 1H, arrow), but that in the dorsomedial subnucleus was eliminated after rhizotomy while that along its lateral border was diminished greatly (Figures 1H, 2I). Subnucleus interpolaris (Figures 1G, 2E,F) showed little immunoreactivity on either the normal or experimental sides of the brainstem. CGRP immunoreactivity in the paratrigeminal nucleus was similar to that for SubP, robust label on the normal side and slightly diminished label on the experimental side (Figure 2D). CGRP immunoreactivity in the MDH was robust in laminae I, II, and V on the normal side, but most of this was generally lost after rhizotomy (Figures 1E,F, 2A–D); numerous fine “dust-like” immunoreactivity was replaced by a sparser, coarse granular reaction product. Dense fibers still were seen occasionally however in lamina I, both dorsally and near the ventral tip of the MDH after rhizotomy, as well as bursts in lamina V, especially near to the spinal dorsal horn. There was very little CGRP immunoreactivity in the nucleus tractus solitarii rostral to the obex on the normal side while that in its caudal half appeared with similar intensity after rhizotomy. CGRP fibers in the dorsal column nuclei were unchanged after rhizotomy.

**Figure 3:**
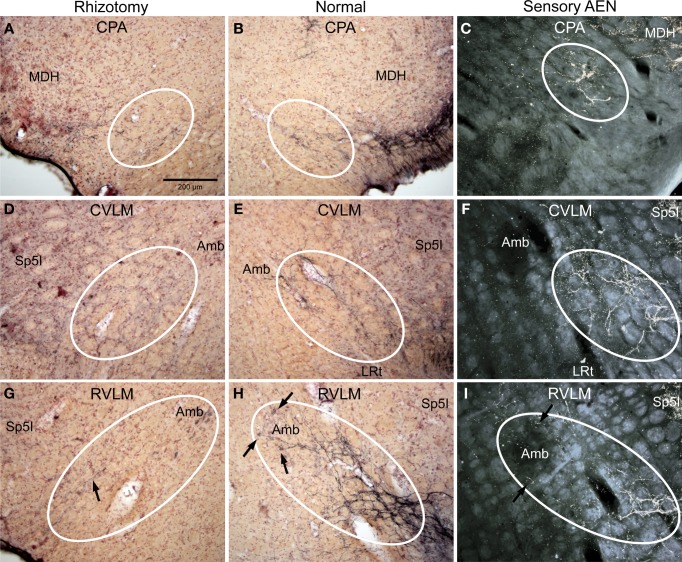
**Photomicrographs of sections illustrating loss of CGRP immunoreactivity in reticular areas after unilateral trigeminal rhizotomy**. Compare the distribution of CGRP fibers on the non-experimental side (**B**,**E**,**H**; Normal) to fibers of the anterior ethmoidal nerve, a branch of the ophthalmic division of the trigeminal, labeled after transganglionic transport (**C**,**F**,**I**; Sensory AEN). Note the striking similarity of these two distributions. The moderate amount of CGRP-labeled fibers in the CPA (**B**, outlined) was reduced but not eliminated after trigeminal rhizotomy **(A)**. However, that to lateral parts of the caudal **(D)** and rostral **(G)** ventrolateral medulla were diminished greatly save for random fibers (**G**, arrow), implicating that these reticular projections of the trigeminal nerve contain CGRP. Also note the projections of CGRP fibers to neurons surrounding the compact formation of nucleus ambiguus (**H**, arrows) similar to those seen after transganglionic transport in the AEN (**I**, arrows). Parasympathetic preganglionic cardiac motor neurons activated during the diving response are distributed similarly. See Figures 1, 2 for abbreviations. All figures are at same magnification.

This sharp contrast of immunostaining for CGRP in the lateral medulla between the experimental and normal sides is best seen in line drawings of sections through the lower brainstem (Figure 2); note the almost total loss of CGRP immunoreactivity in the trigeminal sensory complex, CVLM and RVLM after rhizotomy, and a substantial decrease in the CPA. Unilateral trigeminal rhizotomy decreased CGRP immunoreactivity in the CPA (Figures 2A,B, 3A,B) and almost eliminated immunoreactive CGRP fibers in the CVLM and RVLM when compared to the normal side (Figures 2D–G; areas encircled for CVLM—Figures 3D,E and RVLM—Figures 3G,H). Bands of fibers on the normal side left the ventral part of the spinal trigeminal tract and bifurcated in the RVLM (Figures 2F–H); larger bundles went rostrally to surround neurons juxtaposed to the nucleus ambiguus (Figure 3H; arrows), including its overlap with the facial motor nucleus (Figure 2H), while finer fibers descended into a more ventral part of the RVLM (Figures 2F,G, 5E).

**Figure 4:**
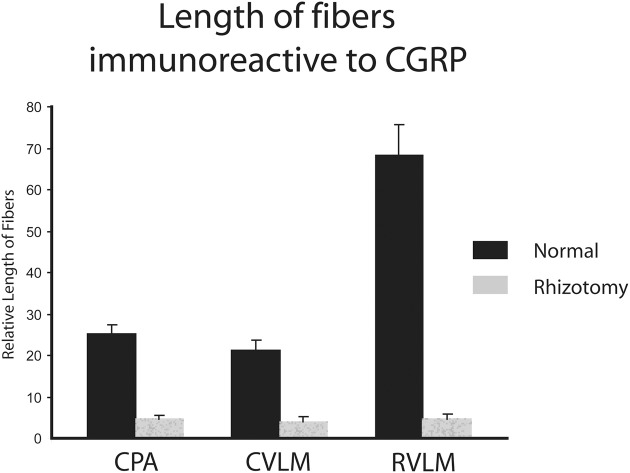
**Bar graphs illustrating the loss of immunostained CGRP fibers in the medullary reticular formation after unilateral trigeminal rhizotomy**. The length of fibers was summed over several sections. Note the marked reduction of fibers in the caudal pressor area (CPA), caudal (CVLM), and rostral (RVLM) ventrolateral medulla after sectioning the trigeminal root, suggesting a primary afferent source from the trigeminal nerve of these fibers.

The character of CGRP immunoreactivity in the reticular formation was found mostly in isolated stained fibers that showed many “swellings,” allowing for easier quantification of fiber length. Thus, labeled fibers in the CPA (boxed area in Figure 1D), caudal ventrolateral reticular formation near the obex (CVLM; boxed area in Figure 1E), and rostral ventrolateral reticular formation (RVLM; boxed area in Figure 1F) were drawn from sections immunostained for CGRP and their length totaled for both normal and rhizotomized sides of the brainstem. Quantification of the length of stained fibers from these cases revealed significant differences (*p* < 0.001) between the two sides (Figure 4), suggesting the source of CGRP fibers in these parts of the reticular formation apparently arise almost exclusively from primary afferent fibers in the trigeminal nerve. Moreover, the character of CGRP immunoreactivity in the reticular formation is remarkably similar to that labeled after transganglionic transport in sensory fibers of the AEN. Note the similar disposition of these fibers in the CPA (Figures 3B,C; encircled), the CVLM (Figures 3E,F; encircled) and RVLM (Figures 3H,I; encircled).

**Figure 5:**
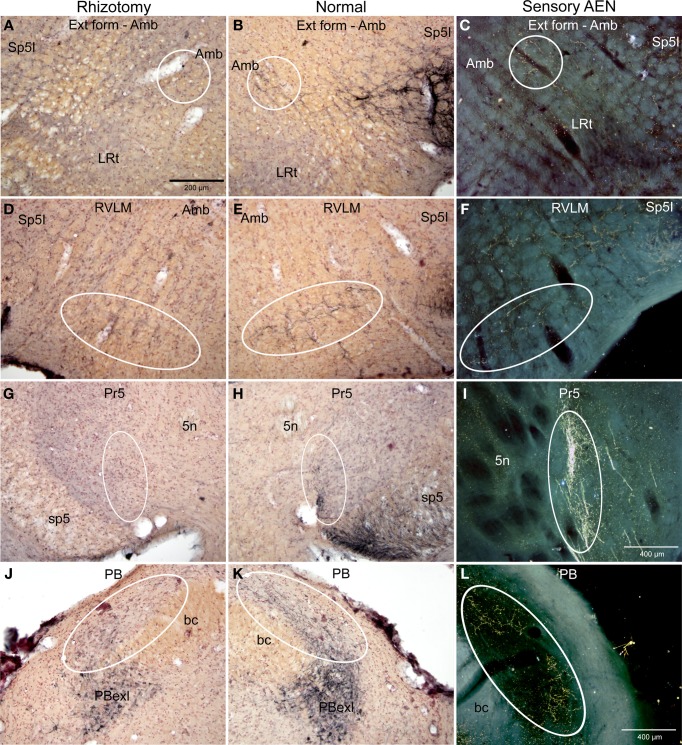
**Photomicrographs of sections illustrating loss of CGRP immunoreactivity in the lateral brainstem after unilateral trigeminal rhizotomy**. Compare the distribution of CGRP fibers on the non-experimental side (**B**,**E**,**H**; Normal) to fibers of the anterior ethmoidal nerve, a branch of the trigeminal, labeled after transganglionic transport (**C**,**F**,**I**; Sensory AEN). Note the striking similarity of these two distributions. The CGRP-labeled fibers juxtaposed to nucleus ambiguus (**B**, encircled; Amb) includes the external formation of Amb where preganglionic cardiac motor neurons are found. Moreover, CGRP-labeled fibers in the ventral RVLM (**E**, encircled), where C1 adrenergic neurons are located, match the distribution of primary afferent fibers carried in the AEN (**F**, encircled). Extratrigeminal primary afferent fibers to the parabrachial complex (**L**, encircled) also match those in normal tissue for CGRP (**K**, encircled). Nearly all such fibers are lost after trigeminal rhizotomy (**A**,**D**,**J**), suggesting the trigeminal origin of CGRP fibers to these nuclei. The principal trigeminal nucleus (Pr5) possesses a tight somatotopy; note the aggregation of CGRP immunoreactive fibers in its ventromedial tip (**H**; encircled) is lost after rhizotomy **(G)**. Also note its disposition mimics that of the anterior ethmoidal nerve (**I**; encircled). Areas encircled **(A–C)**, as well as those marked with ovals **(D–F; G–I; K–L)** mark similar areas of the brainstem. All figures but **I** and **J** are of similar magnification.

Immunolabeled CGRP fibers were also noted near the external formation of the nucleus ambiguus (Figures 2D,E, 5B, encircled), the lateral parts of the parabrachial nucleus (Figures 2K, 5K; encircled), and the more caudal projection to the RVLM on the normal side. Most if not all of these fibers also were eliminated by trigeminal rhizotomy (compare Figures 5A,B; D,E; J,K), suggesting the soma of these fibers also are in the trigeminal ganglion. Moreover, their similarity to extratrigeminal projections of the AEN again is seen (compare Figures 5B,C; E,F; K,L), suggesting their peripheral receptive fields are in the vestibule and anterior mucosa of the nose.

## Discussion

It is well known that numerous primary afferent fibers contain and utilize SubP and CGRP as neurotransmitters/modulators, but few of these fibers project beyond primary relay centers in the brain. Although density of SubP was not quantified, the qualitative appearance of immunoreactive fibers suggested there was a decrease in immunostaining on the lesioned side within the lateral medulla. The loss of CGRP immunoreactivity after rhizotomy was more apparent in all structures studied in the lateral medulla, however, and that in the lateral reticular formation was significantly decreased compared to the non-lesioned side. Moreover, when transganglionic transport of an HRP cocktail applied to the AEN is compared qualitatively to that of CGRP in selected areas of the lateral medulla, the resemblance is remarkable, suggesting that many of these CGRP immunoreactive fibers may travel in this nerve. The CGRP immunoreactive fibers in the lateral reticular formation to the CPA, CVLM, RVLM, and para-ambiguus areas are particularly noteworthy since neurons in these regions significantly influence cardiovascular activity, and may be important in directly influencing these neurons during underwater submersion.

## Technical considerations

Trigeminal rhizotomies are seldom performed but can denervate large areas of neuropil innervated by primary afferent fibers. We waited between 1 and 2 weeks (8–14 days) after rhizotomy since others using a rat model (Sugimoto et al., 1997) suggested that most degeneration had occurred by this time. Unfortunately Sugimoto et al. (1997) only had one rat surviving 2 weeks but three rats surviving 1 week. In a cat model (Tashiro et al., 1991; Stover et al., 1992; Henry et al., 1996) similar trigeminal rhizotomies showed further progressive loss of immunoreactivity for up to 60 days, suggesting the central nervous system may take relatively long periods to ingest degenerating debris. Our results describing degeneration of CGRP immunoreactive fibers in the trigeminal sensory complex were comparable to other descriptions after trigeminal rhizotomy (Tashiro et al., 1991; Bennett-Clarke and Chiaia, 1992; Stover et al., 1992; Henry et al., 1996; Sugimoto et al., 1997), with all studies reporting dramatic loss of immunostaining in most parts of the trigeminal sensory complex with sparing only in parts innervated by peripheral afferent fibers from the facial, glossopharyngeal, vagal, and rostral cervical nerves.

CGRP immunoreactive peripheral neurons emit unmyelinated or thinly myelinated axons (Ishida-Yamamoto et al., 1989; Yamamoto and Senba, 1990), and many studies implicate them in pain behavior. Basbaum and colleagues (Cavanaugh et al., 2011) have shown that transient receptor potential vanilloid-1 (TRPV1) ganglion cells, activated with nociception, are associated preferentially with peptidergic neurons and account for nearly all of the unmyelinated, peptidergic ganglion neurons in the adult. Indeed, 73% of the trigeminal ganglion neurons in their study were CGRP positive. We however emphasize the loss of CGRP immunoreactivity in the lateral reticular formation of the medulla since primary afferent fibers to this region may have direct influence over autonomic activity, especially that regulating cardiovascular behavior. Indeed, TRPV1 immunoreactive fibers, associated exclusively with primary afferent fibers, also are found in the lateral reticular formation (Cavanaugh et al., 2011).

## Peptides in the trigeminal sensory complex

We chose to look at the immunoreactivity to SubP and CGRP in the lateral medulla after trigeminal rhizotomy since these peptides are prominent in primary afferent fibers. While the more subtle loss of SubP after such rhizotomies was difficult to quantify, the loss of CGRP immunoreactivity was more demonstrable. The right and left brainstem sections immunostained for either CGRP or SubP were compared following unilateral trigeminal rhizotomy. After an 8–14 days survival, immunostaining staining for these peptides in the trigeminal sensory nucleus was greatly decreased/eliminated in the spinal trigeminal tract, lamina I, II, and V of the MDH, the subnucleus oralis and principal trigeminal nucleus, substantiating that described following similar rhizotomies reported earlier in rats (Sugimoto et al., 1997) and cats (Tashiro et al., 1991; Bennett-Clarke and Chiaia, 1992; Stover et al., 1992; Henry et al., 1996).

There are numerous neurons containing SubP both in peripheral ganglia as well as the central nervous system, thus discussing changes in immunoreactivity to SubP after rhizotomy is moot. More ganglion cells contain CGRP than SubP however, and SubP and CGRP are co-localized in numerous ganglion cells. Moreover, CGRP is less abundant in central neurons than SubP despite its presence in primary somatosensory relay nuclei and in motor neurons (Kruger et al., 1988), making statements about sensory denervation more compelling. While much CGRP immunostaining in the trigeminal sensory complex was eliminated with trigeminal rhizotomy, several parts retained immunoreactive CGRP fibers. For example, CGRP reactive fibers persisted in laminae I and V near the spinomedullary border. These fibers probably arose from rostral cervical dermatomes that overlap in the MDH (Stover et al., 1992; Sugimoto et al., 1997); Panneton et al. previously have noted primary afferent fibers to these laminae provide only a blurred somatotopy at best (Panneton and Burton, 1981; Panneton, 1991; Panneton et al., 2005a, 2006, 2010c) since numerous peripheral targets provide projections to similar areas of neuropil. Primary afferent fibers in the glossopharyngeal and vagus nerves also invade superficial neuropil of the rostral MDH (Panneton, 1991), including the paratrigeminal nucleus, as well as laminae I and V. Such overlap substantiates that seen in the caudal MDH and spinal dorsal horn, again blurring somatotopy within these laminae. We suspect that these projections maintained immunoreactivity against both CGRP and SubP in the paratrigeminal nucleus and selected parts of lamina I of the MDH after trigeminal rhizotomy.

Loss of CGRP immunoreactivity after rhizotomy in two trigeminal areas particularly emphasize the presence of CGRP in the AEN. Aggregations of CGRP in the ventromedial aspect of the principle trigeminal nucleus (Figures 5G–I) are somatotopically similar to those seen after transganglionic labeling in the AEN (Panneton et al., 2006). Indeed, if one believes a precise somatotopic representation exists in the trigeminal system (e.g., Belford and Killackey, 1979; Waite and De Permentier, 1991; Melzer et al., 1994; Erzurumlu et al., 2010) such overlap predicts unity. Moreover the extensive loss of CGRP immunolabeling in the misplaced substantia gelatinosa of the MDH (Figures 1F, 2D), where AEN fibers terminate, also suggests that numerous fibers within this nerve contain CGRP.

## Peptides in the lateral reticular formation

Of more interest to us however, was the near total loss of CGRP fibers in reticular areas, including the RVLM, CVLM, peri-ambiguus area, and the parabrachial nucleus. This suggests these latter areas in the reticular formation are innervated directly by primary afferent fibers of trigeminal origin. Numerous fibers immunoreactive to CGRP innervate the nasal mucosa (Petersson et al., 1989; Silverman and Kruger, 1989; Stjärne et al., 1989; Finger et al., 1990; Silver et al., 1991; Spit et al., 1993; Matsuda et al., 1994, 1998) which is supplied in part by the AEN. The character of CGRP in these reticular areas mimics the central projections of the AEN, and we suggest that this nerve is the origin of many CGRP immunoreactive fibers in these reticular areas. Indeed, CGRP immunoreactive peripheral neurons emit unmyelinated or thinly myelinated axons (Ishida-Yamamoto et al., 1989; Yamamoto and Senba, 1990), similar to the composition of fibers in the AEN (McCulloch et al., 1999a) supporting our assertion. Moreover, CGRP has been shown to augment reflex activity (Xu et al., 1990; Wiesenfeld et al., 2006) and electrical stimulation of the AEN induces cardiorespiratory responses similar to the diving response (McCulloch et al., 1999a) It would be interesting to determine if ablation of TRPV1 central terminals by intrathecal injections of capsaicin would eliminate the cardiovascular sequelae of AEN stimulation similar to the loss of behavioral responses (Cavanaugh et al., 2009) seen after its intrathecal application in the spinal cord.

## Comparison of reticular projections with those of the AEN

The present data suggests that many of the reticular projections of the trigeminal nerve are CGRP positive, and that these reticular projections highly correlate with the subset provided by the AEN. The AEN is relatively unique among peripheral nerves since its electrical stimulation induces dramatic changes in autonomic rhythmicity including an apnea, drastic reduction in heart rate, and increases in arterial blood pressure (Dutschmann and Herbert, 1996, 1997, 1998b; McCulloch et al., 1999a,b; Rozloznik et al., 2009), responses which mimic those of the mammalian diving response (Panneton et al., 2010a,b, 2012, 2014), a collective of somatoautonomic reflexes aimed to preserve intrinsic oxygen stores (Panneton, 2013). We recently have shown that numerous cardiac motor neurons activated by diving reside in the RVLM, especially surrounding the compact formation of the nucleus ambiguus (Panneton et al., 2014). The appearance of a dense plexus of trigeminal primary afferent fibers immunoreactive to CGRP projecting into similar locations resemble those of the AEN (Figures 3G–I); we suggest these fibers offer direct somatoautonomic projections to cardiac motoneurons. Fewer cardiac motoneurons found more caudally were activated by underwater submersion, but those double-labeled were usually found in the external formation of nucleus ambiguus. It was striking that fibers both immunoreactive to CGRP and sensory from the AEN also project to similar areas (Figures 3D–F, 5A–C).

We have shown previously that neurons just caudal and ventral to the compact formation generate descending projections to sympathetic preganglionic neurons and promote vasoconstriction, resulting in increased arterial blood pressure after nasal stimulation (McCulloch et al., 1999a,b). Immunoreactive CGRP fibers, probably from the AEN (Figures 5D–F), project to similar neuropil where both catecholaminergic and non-catecholaminergic neurons are activated by underwater submersion (Panneton, 2013); we suggest these fibers provide direct somatoautonomic projections to RVLM neurons regulating blood pressure, especially during diving.

A recent report however showed the AEN is not prerequisite for initiation of the diving response in awake voluntarily diving rats, nor in those anesthetized and stimulated nasally (Chotiyanonta et al., 2013), results which differ from their previous report (Rybka and McCulloch, 2006). While our laboratory has emphasized the AEN is important in the diving response, we have never stated this nerve is prerequisite for the diving response to occur. While McCulloch et al. argue that plastic changes occur days after previous bilateral axotomy of the AEN, only a few ganglion cells die after such lesions in adults (Aldskogius and Arvidsson, 1978; Tessler et al., 1985; Rich et al., 1987) and few changes in their central distributions are seen with the light microscope (Sugimoto and Gobel, 1982; Rodin et al., 1983) but some ultrastructural changes may occur (Aldskogius et al., 1985). We saw no regeneration of CGRP in either the reticular formation or trigeminal sensory complex up to 14 days post-rhizotomy, similar to others with even longer survival times (Tashiro et al., 1991; Stover et al., 1992; Henry et al., 1996), but some CGRP filled growth cone-like enlargements in the severed roots have been noted (Henry et al., 1996). While these incongruent results for anesthetized rats stimulated nasally could be explained somewhat by the radical change in doses of anesthesia between these two studies (Rybka and McCulloch, 2006; Chotiyanonta et al., 2013), the retention of the diving response after AEN section is similar to data collected in our laboratory (unpublished) even in unanesthetized decerebrate rats.

The AEN innervates only the *anterior* part of both the septal and lateral walls of the nasal mucosa; posterior mucosa is innervated by small branches emanating from the nasociliary nerve and maxillary division of the trigeminal nerve. Thus, these posterior branches are still intact after sectioning the AEN and potentially could provide input into the CNS to elicit the cardiorespiratory responses during nasal stimulation. Indeed, the dorsal aspect of the misplaced substantia gelatinosa in the rostral MDH, where the maxillary division projects, receives projections from the infraorbital nerve of this division (Panneton, 2013) and could explain results of a previous study (McCulloch et al., 1997) where large injections of amino acid receptor antagonists were placed in the dorsal caudal part of subnucleus interpolaris. In these studies the cardiovascular changes to diving were attenuated but not eliminated.

The maintenance of the diving response in awake behaving rats after AEN section also implicates other paranasal nerves, but probably negates those innervating posterior nasal mucosa since McCulloch and colleagues (Chotiyanonta et al., 2013) provided no indication that the voluntarily diving rats with axotomized AEN's inhaled water over their posterior nasal mucosa during underwater submergence. We suspect filaments of the infraorbital, superior alveolar and nasopalatine nerves (plate 38; Netter, 2003), from the maxillary division and innervating the anterior nasal mucosa, are likely candidates for maintaining the cardiovascular adjustments to diving. Thus, although the AEN is important for diving physiology, it is not necessary for this basic reflex to be induced. Although it has been suggested that cetaceans and pinnepeds with their expanded neocortices may voluntarily control these autonomic parameters with “will” (Panneton, 2013), the maintenance of the response in the lissencepahlic rat after AEN section remains an enigma.

The present study however shows that direct primary afferent projections from the trigeminal nerve invade reticular areas where bradycardia and increased peripheral resistance is generated during underwater submersion. We believe this provides the first instance of a reflex loop bypassing traditional somatic relay nuclei, and implicates the diving response and its respective reflexes as special among reflexes in general. This data fortifies our assertion that the diving response is the most powerful autonomic reflex known.

### Conflict of interest statement

The authors declare that the research was conducted in the absence of any commercial or financial relationships that could be construed as a potential conflict of interest.

## References

[B1] Aldskogius H. Arvidsson J. (1978). Nerve cell degeneration and death in the trigeminal ganglion of the adult rat followng peripheral nerve transection. J. Neurocytol. 7, 229–250 10.1007/BF01217921 650265

[B2] Aldskogius H. J. Arvidsson J. Grant G. (1985). The reaction of primary sensory neurons to peripheral nerve injury with particular emphasis on transganglionic changes. Brain Res. Rev. 10, 27–46 10.1016/0165-0173(85)90006-2 2412661

[B3] Angell James J. E. de Burgh Daly M. (1972). Reflex respiratory and cardiovascular effects of stimulation of receptors in the nose of the dog. J. Physiol. (Lond). 220, 673–696 5016041 10.1113/jphysiol.1972.sp009729 PMC1331676

[B4] Belford G. R. Killackey H. P. (1979). Vibrissae representation in subcortical trigeminal centers of the neonatal rat. J. Comp. Neurol. 183, 305–322 10.1002/cne.901830207 762261

[B5] Bennett-Clarke C. A. Chiaia N. L. (1992). Normal development and effects of neonatal infraorbital nerve damage upon the innervation of the trigeminal brainstem complex by primary afferent fibers containing calcitonin gene-related peptide. J. Comp. Neurol. 324, 282–294 10.1002/cne.903240209 1430333

[B6] Cain W. Murphy C. L. (1980). Interaction between chemoreceptive modalities of odour and irritation. Nature 284, 255–257 10.1038/284255a0 7360255

[B7] Cauna N. Hinderer K. H. Wentges R. T. (1969). Sensory receptor organs of the human nasal respiratory mucosa. Am. J. Anat. 124, 187–210 10.1002/aja.1001240205 5774650

[B8] Cavanaugh D. J. Chesler A. T. Bráz J. M. Shah N. M. Julius D. Basbaum A. I. (2011). Restriction of transient receptor potential vanilloid-1 to the peptidergic subset of primary afferent neurons follows its developmental downregulation in non-peptidergic neurons. J. Neurosci. 31, 10119–10127 10.1523/JNEUROSCI.1299-11.2011 21752988 PMC3147010

[B9] Cavanaugh D. J. Lee H. Lo L. Shields S. D. Zylka M. J. Basbaum A. I. (2009). Distinct subsets of unmyelinated primary sensory fibers mediate behavioral responses to noxious thermal and mechanical stimuli. Proc. Natl. Acad. Sci. U.S.A. 106, 9075–9080 10.1073/pnas.0901507106 19451647 PMC2683885

[B10] Chotiyanonta J. S. DiNovo K. M. McCulloch P. F. (2013). Bilateral sectioning of the anterior ethmoidal nerves does not eliminate the diving response in voluntarily diving rats. Physiol. Rep. 1:e00141 10.1002/phy2.141 24400143 PMC3871456

[B11] Cometto-Muñiz J. E. Cain W. S. (1997). Agonistic sensory effects of airborne chemicals in mixtures: odor, nasal pungency, and eye irritation. Percept. Psychophys. 59, 665–674 10.3758/BF03206014 9259635

[B12] Cometto-Muniz J. E. Cain W. S. Abraham M. H. (1998). Nasal pungency and odor of homologous aldehydes and carboxylic acids. Exp. Brain Res. 118, 180–188 10.1007/s002210050270 9580061

[B13] Cometto-Muniz J. E. Cain W. S. Abraham M. H. Gola J. M. R. (2001). Ocular and nasal trigeminal detection of butyl acetate and toluene presented singly and in mixtures. Toxicol. Sci. 63, 233–244 10.1093/toxsci/63.2.233 11568367

[B14] Drummond P. C. Jones D. R. (1979). The initiation and maintenance of bradycardia in a diving mammal, the muskrat, *Ondatra zibethica*. J. Physiol. (Lond). 290, 253–271 469759 10.1113/jphysiol.1979.sp012770 PMC1278834

[B15] Dutschmann M. Herbert H. (1996). The Kölliker-Fuse nucleus mediates the trigeminally induced apnoea in the rat. Neuroreport 7, 1432–1436 10.1097/00001756-199605310-00022 8856692

[B16] Dutschmann M. Herbert H. (1997). Fos expression in the rat parabrachial and Kölliker-Fuse nuclei after electrical stimulation of the trigeminal ethmoidal nerve and water stimulation of the nasal mucosa. Exp. Brain Res. 117, 97–110 10.1007/s002210050203 9386008

[B17] Dutschmann M. Herbert H. (1998a). The medial nucleus of the solitary tract mediates the trigeminally evoked pressor response. Neuroreport 9, 1053–1057 9601666

[B18] Dutschmann M. Herbert H. (1998b). NMDA and GABAA receptors in the rat Kolliker-Fuse area control cardiorespiratory responses evoked by trigeminal ethmoidal nerve stimulation. J. Physiol. (Lond). 510, 793–804 10.1111/j.1469-7793.1998.793bj.x 9660894 PMC2231078

[B19] Erzurumlu R. S. Murakami Y. Rijli F. M. (2010). Mapping the face in the somatosensory brainstem. Nat. Rev. Neurosci. 11, 252–263 10.1038/nrn2804 20179712 PMC3545448

[B20] Finger T. E. St. Jeor V. L. Kinnamon J. C. Silver W. L. (1990). Ultrastructure of substance P- and CGRP-immunoreactive nerve fibers in the nasal epithelium of rodents. J. Comp. Neurol. 294, 293–305 10.1002/cne.902940212 1692045

[B21] Gieroba Z. J. Yu Y.-H. Blessing W. W. (1994). Vasoconstriction induced by inhalation of irritant vapour is associated with appearance of Fos protein in C1 catecholamine neurons in rabbit medulla oblongata. Brain Res. 636, 157–161 10.1016/0006-8993(94)90192-9 7908852

[B22] Green B. G. Lawless H. T. (1991). The psychophysics of somatosensory chemoreception in the nose and mouth, in Smell and Taste in Health and Disease, eds Getchell T. V. Dory R. L. Bartoshuk L. M. Snow J. B. (New York, NY: Raven Press), 235–253

[B23] Handwerker H. O. Kobal G. (1993). Psychophysiology of experimentally induced pain. Physiol. Rev. 73, 639–671 8332641 10.1152/physrev.1993.73.3.639

[B24] Henry M. A. Johnson L. R. Nousek-Goebl N. Westrum L. E. (1996). Light microscopic localization of calcitonin gene-related peptide in the normal feline trigeminal system and following retrogasserian rhizotomy. J. Comp. Neurol. 365, 526–540 10.1002/(SICI)1096-9861(19960219)365:4<526::AID-CNE2>3.0.CO;2-6 8742300

[B25] Hollandsworth M. P. DiNovo K. M. McCulloch P. F. (2009). Unmyelinated fibers of the anterior ethmoidal nerve of the rat co-localize with neurons in the medullary dorsal horn and ventrolateral medulla activated by nasal stimulation. Brain Res. 1298, 131–144 10.1016/j.brainres.2009.08.077 19732757 PMC2760627

[B26] Hummel T. Mohammadian P. Marchl R. Kobal G. Loetsch J. (2003). Pain in the trigeminal system: irritation of the nasal mucosa using short- and long-lasting stimuli. Int. J. Psychophysiol. 47, 147–158 10.1016/S0167-8760(02)00150-2 12568945

[B27] Ichikawa H. Mitani S. Hijiya H. Nakago T. Jacobowitz D. M. Sugimoto T. (1993). Calretinin-immunoreactivity in trigeminal neurons innervating the nasal mucosa of the rat. Brain Res. 629, 231–238 10.1016/0006-8993(93)91325-M 8111627

[B28] Ishida-Yamamoto A. Senba E. Tohyama M. (1989). Distribution and fine structure of calcitonin gene-related peptide-like immunoreactive nerve fibers in the rat skin. Brain Res. 491, 93–101 10.1016/0006-8993(89)90090-5 2788478

[B29] Kratschmer F. (2001). On reflexes from the nasal mucous membrane on respiration and circulation. Respir. Physiol. 127, 93–104 10.1016/S0034-5687(01)00234-1 11504582

[B30] Kruger L. Sternini C. Brecha N. C. Mantyh P. W. (1988). Distribution of calcitonin gene-related peptide immunoreactivity in relation to the rat central somatosensory projection. J. Comp. Neurol. 273, 149–162 10.1002/cne.902730203 3047185

[B31] Lucier G. E. Egizii R. (1989). Characterization of cat nasal afferents and brain stem neurones receiving ethmoidal input. Exp. Neurol. 103, 83–89 10.1016/0014-4886(89)90189-1 2536331

[B32] Marfurt C. F. (1981). The somatotopic organization of the cat trigeminal ganglion as determined by the horseradish peroxidase technique. Anat. Rec. 201, 105–118 10.1002/ar.1092010113 6975586

[B33] Marfurt C. F. Rajchert D. M. (1991). Trigeminal primary afferent projections to “non-trigeminal” areas of the rat central nervous system. J. Comp. Neurol. 303, 489–511 10.1002/cne.903030313 1706735

[B34] Matsuda H. Kusakabe T. Hayashida Y. Furukawa M. Kawakami T. Takenaka T. (1998). Substance P- and calcitonin gene-related peptide-containing nerve fibers in the nasal mucosa of chronically hypoxic rats. Brain Res. Bull. 45, 563–569 10.1016/S0361-9230(97)00450-4 9566499

[B35] Matsuda H. Tsukuda M. Kadota T. Kusunoki T. Kishida R. (1994). Coexistence of galanin and substance P in the mouse nasal mucosa, including the vomeronasal organ. Neurosci. Lett. 173, 55–58 10.1016/0304-3940(94)90148-1 7524000

[B36] McCulloch P. F. Faber K. M. Panneton W. M. (1999a). Electrical stimulation of the anterior ethmoidal nerve produces the diving response. Brain Res. 830, 24–31 10.1016/S0006-8993(99)01374-8 10350556

[B37] McCulloch P. F. Ollenberger G. P. Bekar L. K. West N. H. (1997). Trigeminal and chemoreceptor contributions to bradycardia during voluntary dives in rats. Am. J. Physiol. 273, R814–R822 9277573 10.1152/ajpregu.1997.273.2.R814

[B38] McCulloch P. F. Panneton W. M. Guyenet P. G. (1999b). The rostral ventrolateral medulla mediates the sympathoactivation produced by chemical stimulation of the nasal mucosa. J. Physiol. (Lond). 516, 471–484 10.1111/j.1469-7793.1999.0471v.x 10087346 PMC2269263

[B39] McRitchie R. J. White S. W. (1974). Role of trigeminal, olfactory, carotid sinus and aortic nerves in the respiratory and circulatory response to nasal inhalation of cigarette smoke and other irritants in the rabbit. Aust. J. Exp. Biol. Med. Sci. 52, 127–140 10.1038/icb.1974.10 4827813

[B40] Melzer P. Welker E. Dörfl J. Van der Loos H. (1994). Maturation of the neuronal metabolic response to vibrissa stimulation in the developing whisker-to-barrel pathway of the mouse. Dev. Brain Res. 77, 227–250 10.1016/0165-3806(94)90199-6 8174231

[B41] Netter F. H. (2003). Atlas of Human Anatomy. Teterboro, NJ: Icon Learning Systems

[B42] Panneton W. M. (1990). Controlled bradycardia induced by nasal stimulation in the muskrat, *Ondatra zibethicus*. J. Auton. Nerv. Syst. 30, 253–264 10.1016/0165-1838(90)90257-J 2229892

[B43] Panneton W. M. (1991). Primary afferent projections from the upper respiratory tract in the muskrat. J. Comp. Neurol. 308, 51–65 10.1002/cne.903080106 1714922

[B44] Panneton W. M. (2013). The mammalian diving response: an enigmatic reflex to preserve life? Physiology 28, 284–297 10.1152/physiol.00020.2013 23997188 PMC3768097

[B45] Panneton W. M. Anch A. M. Panneton W. M. Gan Q. (2014). Parasympathetic preganglionic cardiac motoneurons labeled after voluntary diving. Front. Physiol. 5:8 10.3389/fphys.2014.00008 24478721 PMC3904087

[B46] Panneton W. M. Burton H. (1981). Corneal and periocular representation within the trigeminal sensory complex in the cat studied with transganglionic transport of horseradish peroxidase. J. Comp. Neurol. 199, 327–344 10.1002/cne.901990303 7263952

[B47] Panneton W. M. Gan Q. Dahms T. E. (2010a). Cardiorespiratory and neural consequences of rats brought past their aerobic dive limit. J. Appl. Physiol. 109, 1256–1269 10.1152/japplphysiol.00110.2010 20705947 PMC2971699

[B48] Panneton W. M. Gan Q. Juric R. (2005a). The central termination of sensory fibers from nerves to the gastrocnemius muscle of the rat. Neuroscience 134, 175–187 10.1016/j.neuroscience.2005.02.032 15953682

[B49] Panneton W. M. Gan Q. Juric R. (2005b). CGRP in the reticular formation is from primary afferent fibers. Neurosci. Abstr. 30 1629401

[B50] Panneton W. M. Gan Q. Juric R. (2006). Brainstem projections from recipient zones of the anterior ethmoidal nerve in the medullary dorsal horn. Neuroscience 141, 889–906 10.1016/j.neuroscience.2006.04.055 16753263

[B51] Panneton W. M. Gan Q. Juric R. (2010b). The rat: a laboratory model for studies of the diving response. J. Appl. Physiol. 108, 811–820 10.1152/japplphysiol.00600.2009 20093670 PMC2853196

[B52] Panneton W. M. Gan Q. Le J. Livergood R. S. Clerc P. Juric R. (2012). Activation of brainstem neurons by underwater diving in the rat. Front. Physiol. 3:111 10.3389/fphys.2012.00111 22563319 PMC3342523

[B53] Panneton W. M. Hsu H. Gan Q. (2010c). Distinct central representations for sensory fibers innervating either the conjunctiva or cornea of the rat. Exp. Eye Res. 90, 388–396 10.1016/j.exer.2009.11.018 20004193 PMC2824013

[B54] Paxinos G. Watson C. (1998). The Rat Brain in Stereotaxic Coordinates. San Diego, CA: Academic Press

[B55] Petersson G. Malm L. Ekman R. Håkanson R. (1989). Capsaicin evokes secretion of nasal fluid and depletes substance P and calcitonin gene-related peptide from the nasal mucosa in the rat. Br. J. Pharmacol. 98, 930–936 10.1111/j.1476-5381.1989.tb14623.x 2480171 PMC1854768

[B56] Rich K. M. Luszczynski J. R. Osborne P. A. Johnson E. M. Jr. (1987). Nerve growth factor protects adult sensory neurons from cell death and atrophy caused by nerve injury. J. Neurocytol. 16, 261–268 3625240 10.1007/BF01795309

[B57] Rodin B. E. Sampogna S. L. Kruger L. (1983). An examination of intraspinal sprouting in dorsal root axons with the tracer horseradish peroxidase. J. Comp. Neurol. 215, 187–198 10.1002/cne.902150206 6853772

[B58] Rozloznik M. Paton J. F. R. Dutschmann M. (2009). Repetitive paired stimulation of nasotrigeminal and peripheral chemoreceptor afferents cause progressive potentiation of the diving bradycardia. Am. J. Physiol. Regul. Integr. Comp. Physiol. 296, R80–R87 10.1152/ajpregu.00806.2007 18987289

[B59] Rybka E. J. McCulloch P. F. (2006). The anterior ethmoidal nerve is necessary for the initiation of the nasopharyngeal response in the rat. Brain Res. 1075, 122–132 10.1016/j.brainres.2005.12.112 16466647

[B60] Schaefer M. L. Boettger B. Silver W. L. Finger T. E. (2002). Trigeminal collaterals in the nasal epithelium and olfactory bulb: a potential route for direct modulation of olfactory information by trigeminal stimuli. J. Comp. Neurol. 444, 221–226 10.1002/cne.10143 11840476

[B61] Sekizawa S. Tsubone H. (1994). Nasal receptors responding to noxious chemical irritants. Respir. Physiol. 96, 37–48 10.1016/0034-5687(94)90104-X 8023019

[B62] Sekizawa S. I. Tsubone H. (1996). Nasal mechanoreceptors in guinea pigs. Respir. Physiol. 106, 223–230 10.1016/S0034-5687(96)00085-0 9017840

[B63] Silver W. L. Farley L. G. Finger T. E. (1991). The effects of neonatal capsaicin administration on trigeminal nerve chemoreceptors in the rat nasal cavity. Brain Res. 561, 212–216 10.1016/0006-8993(91)91597-T 1724948

[B64] Silverman J. D. Kruger L. (1989). Calcitonin-gene-related-peptide-immunoreactive innervation of the rat head with emphasis on specialized sensory structures. J. Comp. Neurol. 280, 303–330 10.1002/cne.902800211 2784449

[B65] Spit B. J. Bretschneider F. Hendriksen E. G. J. Kuper C. F. (1993). Ultrastructure of free nerve endings in respiratory and squamous epithelium on the rat nasal septum. Cell Tissue Res. 274, 329–335 10.1007/BF00318751 7505720

[B66] Stjärne P. Lundblad L. Änggård A. Hökfelt T. Lundberg J. M. (1989). Tachykinins and calcitonin gene-related peptide: co-existence in sensory nerves of the nasal mucosa and effects on blood flow. Cell Tissue Res. 256, 439–446 10.1007/BF00225591 2787209

[B67] Stover J. D. Schwab C. A. Mathews M. A. (1992). Selective deaffferentation of convergent inputs to trigeminal subnucleus caudalis: effects on calcitonin gene-related peptide distribution. Somatosens. Mot. Res. 9, 107–130 10.3109/08990229209144766 1502861

[B68] Sugimoto T. Fujiyoshi Y. Xiao C. He Y. F. Ichikawa H. (1997). Central projection of calcitonin gene-related peptide (CGRP)-and substance P (SP)-immunoreactive trigeminal primary neurons in the rat. J. Comp. Neurol. 378, 425–442 10.1002/(SICI)1096-9861(19970217)378:3<425::AID-CNE9>3.0.CO;2-5 9034901

[B69] Sugimoto T. Gobel S. (1982). Primary neurons maintain their central axonal arbors in the spinal dorsal horn following peripheral nerve injury: an anatomical analysis using transganglionic transport of horseradish peroxidase. Brain Res. 248, 377–381 10.1016/0006-8993(82)90598-4 7139284

[B70] Sun W. Panneton W. M. (2002). The caudal pressor area of the rat: its precise location and projections to the ventrolateral medulla. Am. J. Physiol. Regul. Integr. Comp. Physiol. 283, R768–R778 10.1152/ajpregu.00184.2002 12185012

[B71] Tashiro T. Takahashi T. Satoda T. Matsushima R. Uemura-Sumi M. Mizuno N. (1991). Distibution of axons showing calcitonin gene-related peptide and/or substance P-like immunoreactivity in the sensory trigeminal nuclei of the cat. Neurosci. Res. 11, 119–133 10.1016/0168-0102(91)90050-9 1717903

[B72] Tessler A. Himes B. T. Krieger N. R. Murray M. Goldberger M. E. (1985). Sciatic nerve transection produces death of dorsal root ganglion cells and reversible loss of substance P in spinal cord. Brain Res. 332, 209–218 10.1016/0006-8993(85)90590-6 2581651

[B73] Thürauf N. Hummel T. Kettenmann B. Kobal G. (1993). Nociceptive and reflexive responses recorded from the human nasal mucosa. Brain Res. 629, 293–299 10.1016/0006-8993(93)91333-N 8111632

[B74] Viana F. (2011). Chemosensory properties of the trigeminal system. ACS Chem. Neurosci. 2, 38–50 10.1021/cn100102c 22778855 PMC3369707

[B75] Waite P. M. E. De Permentier P. (1991). The rat's postero-orbital sinus hair: I. Brainstem projections and the effect of infraorbital nerve section at different ages. J. Comp. Neurol. 312, 325–340 10.1002/cne.903120302 1660903

[B76] Wallois F. Macron J. M. Jounieaux V. Duron B. (1991). Trigeminal nasal receptors related to respiration and to various stimuli in cats. Respir. Physiol. 85, 111–125 10.1016/0034-5687(91)90010-G 1947448

[B77] Wallois F. Macron J. M. Jounieaux V. Duron B. (1992). Influence of trigeminal nasal afferents on bulbar respiratory neuronal activity. Brain Res. 599, 105–116 10.1016/0006-8993(92)90857-6 1493542

[B78] Wiesenfeld H. Z. Xu X.-J. Hakanson R. Feng D. M. Folkers K. Kristensson K. (2006). On the role of substance P, galanin, vasoactive intestinal peptide, and calcitonin gene-related peptide in mediation of spinal reflex excitability in rats with intact and sectioned peripheral nerves. Ann. N.Y. Acad. Sci. 632, 198–211 10.1111/j.1749-6632.1991.tb33108.x 1719866

[B79] Xu X.-J. Weisenfeld-Hallin Z. Villar M. J. Fahrenkrug J. Hökfelt T. (1990). On the role of galanin, substance P and other neuropeptides in primary sensory neurons of the rat: studies on spinal reflex excitability and peripheral axotomy. Eur. J. Neurosci. 2, 733–743 10.1111/j.1460-9568.1990.tb00464.x 12106274

[B80] Yamamoto A. I. Senba E. (1990). Cell types and axonal sizes of calcitonin gene-related peptide-containing primary sensory neurons of the rat. Brain Res. Bull. 24, 759–764 10.1016/0361-9230(90)90136-N 2372695

